# Barriers to stroke treatment: The price of long-distance from thrombectomy centers

**DOI:** 10.1177/15910199241278036

**Published:** 2024-09-05

**Authors:** Olav Søvik, Halvor Øygarden, Arnstein Tveiten, Martin Wilhelm Kurz, Kathinka Dæhli Kurz, Pål Johan Stokkeland, Hanne Brit Hetland, Hege Langli Ersdal, Per Kristian Hyldmo

**Affiliations:** 1 621781 Faculty of Health Sciences, University of Stavanger, Stavanger, Norway; 2 Department of Research, 60513 Sørlandet Hospital, Kristiansand, Norway; 3 Institute of Clinical Medicine, 6305 University of Oslo, Oslo, Norway; 4 Department of Neurology, 60513 Sørlandet Hospital, Kristiansand, Norway; 5 Department of Neurology, Neuroscience Research Group, 60496 Stavanger University Hospital, Stavanger, Norway; 6 Department of Clinical Science, 1658 University of Bergen, Bergen, Norway; 7 Department of Radiology, 60496 Stavanger University Hospital, Stavanger, Norway; 8 Department of Electrical Engineering and Computer Science, University of Stavanger, Stavanger, Norway; 9 Department of Radiology, Sørlandet Hospital, Kristiansand, Norway; 10 Department of Research, Section of Biostatistics, 60496 Stavanger University Hospital, Stavanger, Norway; 11 Department of Simulation-based Learning, 60496 Stavanger University Hospital, Stavanger, Norway

**Keywords:** Thrombectomy, large vessel occlusion, missed diagnosis, stroke units, distance

## Abstract

**Background:**

Endovascular thrombectomy, the preferred treatment for acute large-vessel occlusion stroke, is highly time-dependent. Many patients live far from thrombectomy centers due to large geographical variations in stroke services. This study aimed to explore the consequences of long transport distance on the proportion of thrombectomy-eligible patients who underwent thrombectomy, the clinical outcomes with or without thrombectomy, the timelines for patients transported, and the diagnostic accuracy of large-vessel occlusion in primary stroke centers.

**Methods:**

We conducted a retrospective observational study in a county with only primary stroke centers, ∼ 300 km from the nearest thrombectomy center. All stroke patients admitted over a year were retrieved from the Norwegian Stroke Registry. A neuroradiologist identified all computed tomography images with large-vessel occlusions. A panel determined whether these patients had a corresponding clinical indication for thrombectomy.

**Results:**

A total of 50% of the eligible patients did not receive thrombectomy. These patients had a significantly higher risk of severe disability or death compared to the patients who underwent thrombectomy. The median time from computed tomography imaging at the primary stroke center to arrival at the thrombectomy center was over 3 hours. Additionally, 30% of the large-vessel occlusions were initially undiagnosed, and half of these patients had a corresponding clinical indication for thrombectomy.

**Conclusions:**

In a county with a long transport distance to a thrombectomy center, a high proportion of eligible patients did not undergo thrombectomy, negatively impacting clinical outcomes. The transport time was considerable. A high rate of large-vessel occlusions was initially not diagnosed.

## Introduction

Endovascular thrombectomy (EVT) is a highly effective treatment for acute ischemic stroke caused by a large-vessel occlusion (LVO).^1^ EVT is recommended to be performed in comprehensive stroke centers with high patient volumes, suitable equipment, and expertise, typically located in densely populated areas.^2^ However, due to geographical variation and uneven distribution of thrombectomy centers, many LVO stroke patients live far from these centers.^3^ EVT is a time-critical treatment.^4^ As time elapses, the proportion of LVO stroke patients eligible for EVT declines due to the progression of irreversible brain infarction.^5^ The likelihood of good clinical outcomes also diminishes rapidly over time.^6^

In a region with only primary stroke centers, ∼ 300 km from the nearest comprehensive thrombectomy center, we conducted a study to explore the challenges and consequences of not having a local EVT treatment option. This study region provides a unique opportunity to understand the impact of long-distance transfers on the eligibility and outcomes of EVT for stroke patients.

The aims of the study were to determine the proportion of EVT-eligible patients who underwent EVT, analyze patient characteristics and clinical outcomes with or without EVT, investigate the timelines for patients transported to the comprehensive stroke center, and assess the diagnostic accuracy of LVO in the primary stroke centers.

## Methods

### Context

All emergency hospitals in Norway operate within a common public health system. Sørlandet Hospital Health Trust (SSHF) serves a population of ∼ 310,000 people in Agder County, located in southern Norway. During the study period in 2018, acute stroke patients were initially admitted to one of the three primary stroke centers within the county. LVO stroke patients considered eligible for EVT were then transported ∼ 300 km, mostly by helicopter, to Oslo University Hospital, the nearest comprehensive stroke center. The current study serves as a baseline prior to the establishment of a local EVT-capable stroke center in Agder County in 2019. The Regional Committee for Medical and Health Research approved the study.

### Data collection

We accessed high-quality data from the Norwegian Stroke Registry, a national quality registry for stroke treatment that records clinical and technical outcomes of all administered treatments.^7^ The registry's coverage is excellent, accurate, and nearly complete, making it a reliable source of data for stroke research.^8–10^ Supplemental data were collected from electronic patient records when data from the Norwegian Stroke Registry were incomplete or missing.

### Study design

The study included all adult patients in Agder County admitted with ischemic stroke in 2018, the last year without a local EVT-capable stroke center. There was no upper age limit. Patients with symptom onset more than 24 h before admission were excluded, as the evidence for EVT is limited to 24 h.^11^ An external neuroradiologist, blinded to the therapeutic decisions and clinical course of the patients, reviewed all computed tomography (CT) angiograms, CT perfusions, and non-contrast CTs to identify LVO strokes with radiological indications for EVT.

A panel of three neurologists and one interventional radiologist then assessed clinical data from the Norwegian Stroke Registry and patient records to determine whether the LVO patients also had clinical indications for EVT. They also checked if LVO had been diagnosed correctly at the primary stroke center at admission, whether the patients were transported to the comprehensive stroke center, and whether EVT was performed.

Clinical EVT indication was determined by criteria commonly used in 2018,^12^ recommending treatment initiation within 6 h of symptom onset. EVT indication in an extended time window up to 24 h^11,13^ was not implemented in Agder County at this time. If there was an indication for acute carotid artery stenting due to critical cerebral hypoperfusion, the patient was classified as having a clinical EVT indication.

### Patient characteristics

The baseline characteristics included age, sex, comorbidities, antithrombotic medication, smoking status, the National Institute of Health Stroke Scale (NIHSS)^14^ score, the pre-stroke modified Rankin scale (mRS) score,^15^ and whether intravenous thrombolysis treatment was administered. From the patient's CT images, the external neuroradiologist recorded the Alberta Stroke Program Early CT Score (ASPECTS)^16^ or posterior ASPECTS,^17^ and the intracranial arterial occlusion site.

### Outcomes

The outcomes included the NIHSS score after 24 h and the mRS score after 3 months for the patients with clinical indication for EVT. For those who underwent EVT, we registered the Modified Thrombolysis in Cerebral Infarction (mTICI) score^18^ and symptomatic intracranial hemorrhage (SICH),^19^ defined as bleeding causing an increase in NIHSS score by four or more points or leading to death within 24 h of treatment. The Expanded Treatment in Cerebral Infraction (eTICI)^20^ score was not recorded, as it was not yet implemented in 2018.

### Timelines for transported patients

For patients transported to the comprehensive stroke center, we recorded relevant time points related to their transfer. These included symptom onset, arrival at the primary stroke center, initial CT imaging, arrival at the comprehensive stroke center, and any repeated imaging at the comprehensive stroke center. For the patients who underwent EVT, we also recorded the time points for arterial puncture and brain reperfusion.

### Statistical analysis

Statistical analyses were performed using SPSS Statistics version 29 (IBM Cooperation, Armonk, NY, USA). Numerical results were described with medians and quartiles. Differences between groups were examined using the Mann–Whitney *U*-test, chi-square test, or Fisher's exact test. A significance level of 0.05 was used to determine statistical significance.

## Results

### The proportion of EVT-eligible patients receiving EVT and diagnostic accuracy of LVO

**Figure 1.**
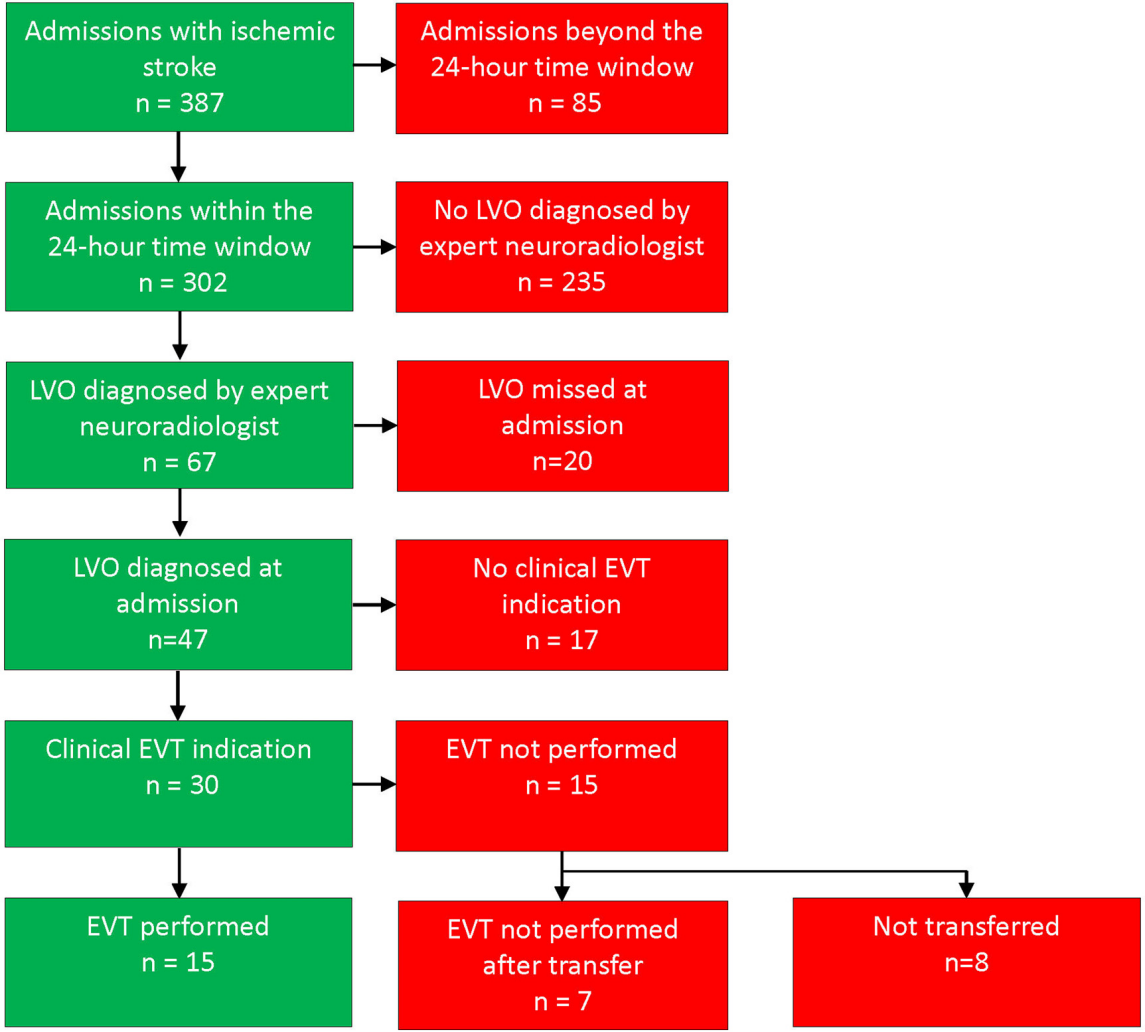
Vascular findings and EVT for Agder County patients admitted with ischemic stroke in 2018. EVT: endovascular thrombectomy; LVO: large-vessel occlusion. A total of 30 patients were correctly diagnosed with LVO on initial assessment with a corresponding clinical EVT indication, 15 of these patients (50%) had EVT performed. A total of 20 of 67 patients (30%) had a missed LVO diagnosis on initial assessment.

In 2018, there were 387 patients admitted with acute ischemic stroke in Agder County. Only 15 (3.9%) of these actually underwent EVT (Figure 1).

Eighty-five were excluded due to symptom onset more than 24 h prior to hospital arrival. Of the remaining patients, 67 had LVO radiologically eligible for EVT. The LVO diagnosis was initially missed in 20 of these patients (30%).

Among the 30 patients correctly diagnosed with LVO and eligible for EVT, there was a notable gender disparity. Specifically, there were significantly fewer women in the EVT performed group and more women in the EVT not performed group (*p* = 0.028, Table 1). Regarding other baseline demographical data, intravenous thrombolytic treatment, ASPECT scores, and thrombus localization, no significant differences were found.

**Table 1.** Patient characteristics.

Baseline characteristics	EVT performed ***n*** = 15	EVT not performed ***n*** = 15	***p***-value
**Age** – median (IQR)	**73 (70–77)**	**79 (67–86)**	**0.187**
**Female sex** – ***n*** (%)	**4 (26.7%)**	**10 (66.7%)**	**0.028**
**Comorbidity** – ***n*** (%)			
Hypertension	**13 (86.7%)**	**13 (86.7%)**	**1.000**
Hyperlipidemia	**9 (60.0%)**	**4 (26.7%)**	**0.065**
Atrial fibrillation	**8 (53.3%)**	**6 (40.0%)**	**0.464**
Diabetes mellitus	**4 (26.7%)**	**4 (26.7%)**	**1.000**
Previous myocardial infarction	**2 (13.3%)**	**0 (0%)**	**0.483**
Previous stroke	**2 (13.3%)**	**6 (40.0%)**	**0.215**
Previous TIA	**1 (6.7%)**	**1 (6.7%)**	**1.000**
**Pre-stroke medication** – ***n*** (%)			
Pre-stroke antiplatelets^a^	**8 (53.3%)**	**6 (40.0%)**	**0.464**
Pre-stroke anticoagulants^b^	**3 (20.0%)**	**3 (20.0%)**	**1.000**
**Pre-stroke known smoker** – ***n*** (%)	**2 (13.3%)**	**3 (20.0%)**	**1.000**
**Pre-stroke mRS > 2** – ***n*** (%)	**1 (6.7%)**	**4 (26.7%)**	**0.330**
**IV thrombolysis treatment** – ***n*** (%)	**12 (80.0%)**	**7 (46.7%)**	**0.058**
**ASPECTS** – median (IQR)			
Anterior	**10 (8–10)**	**9 (7–10)**	**0.146**
Posterior	**10 (10-10)**	**10 (10-10)**	**1.000**
**Thrombus localization** – ***n*** (%)			
ICA	**1 (6.7%)**	**5 (33.3%)**	**0.169**
ICA** **+** **M1	**2 (13.3%)**	**1 (6.7%)**	**1.000**
Carotid T	**0 (0%)**	**0 (0%)**	
M1	**7 (46.7%)**	**5 (33.3%)**	**0.456**
M2	**3 (20.0%)**	**2 (13.3%)**	**1.000**
ACA	**0 (0%)**	**0 (0%)**	
PCA	**0 (0%)**	**0 (0%)**	
Basilar artery	**2 (13.3%)**	**1 (6.7%)**	**1.000**
Vertebral artery	**0 (0%)**	**0 (0%)**	

EVT: endovascular thrombectomy; *n*: number of patients; IQR: interquartile range; TIA: transient ischemic attack; mRS: modified Rankin score; IV: intravenous; ASPECTS: Alberta Stroke Program Early CT Score; ICA: internal carotid artery; Carotid T: terminal bifurcation of the internal carotid artery; M1: first segment of the middle cerebral artery, M2: second segment of the middle cerebral artery; ACA: anterior cerebral artery; PCA: posterior cerebral artery.

a Acetylsalicylic acid, clopidogrel, or dipyridamole.

b Warfarin or other per oral anticoagulation.

Of these 30 patients, eight (27%) were discussed with the comprehensive stroke center, but not accepted for transfer. Twenty-two patients were transported to the comprehensive stroke center. Seven of these transported patients (32%) did not undergo EVT after transport due to low NIHSS for one patient, and small penumbra or large established infarction in the six others. Fifteen of the 30 eligible patients (50%) underwent EVT after transport.

One patient had a delayed LVO stroke diagnosis but underwent EVT outside the 24 h time window on vital indication and is included as EVT performed.

Of the 20 patients with missed LVO diagnosis at the primary stroke centers, 10 (50%) had a clinical indication for EVT. Some patients had multiple admissions with ischemic stroke in 2018, but none had LVO more than once. Details are listed in Supplemental Table S1.

### Clinical outcomes

For the 15 patients who received EVT, the median NIHSS score was 16 on admission at the primary stroke center and 9 after 24 h. The 15 eligible patients who did not receive EVT had a median NIHSS score of 17 both at admission and after 24 h (Table 2, *p* = 0.021).

**Table 2.** NIHSS score.

NIHSS median (IQR)	EVT performed ***n*** = 15	EVT not performed ***n*** = 15
At admission	**16 (12–20)**	**17 (12–24)**
After 24 h	**9 (2–16)**	**17 (10–23)**
Difference^a^	**−7 (−12–0)**	**2 (−2–9)**

NIHSS: National Institute of Health Stroke Scale; IQR: interquartile range; *n*: number of patients; EVT: endovascular thrombectomy.

a Negative value represents the clinical improvement and positive value represents the clinical worsening.

Three patients were intubated and sedated after 24 h and have therefore received a NIHSS score of 39.^21^

The difference was significantly worse for the EVT not performed group (*p* = 0.021).

After EVT, the mTICI score was graded as 2b in five patients (33%) and as mTICI grade 3 in 10 patients (67%). Asymptomatic subarachnoid hemorrhage was detected after EVT in two patients, but none of them suffered from symptomatic intracranial bleeding.

**Figure 2.**
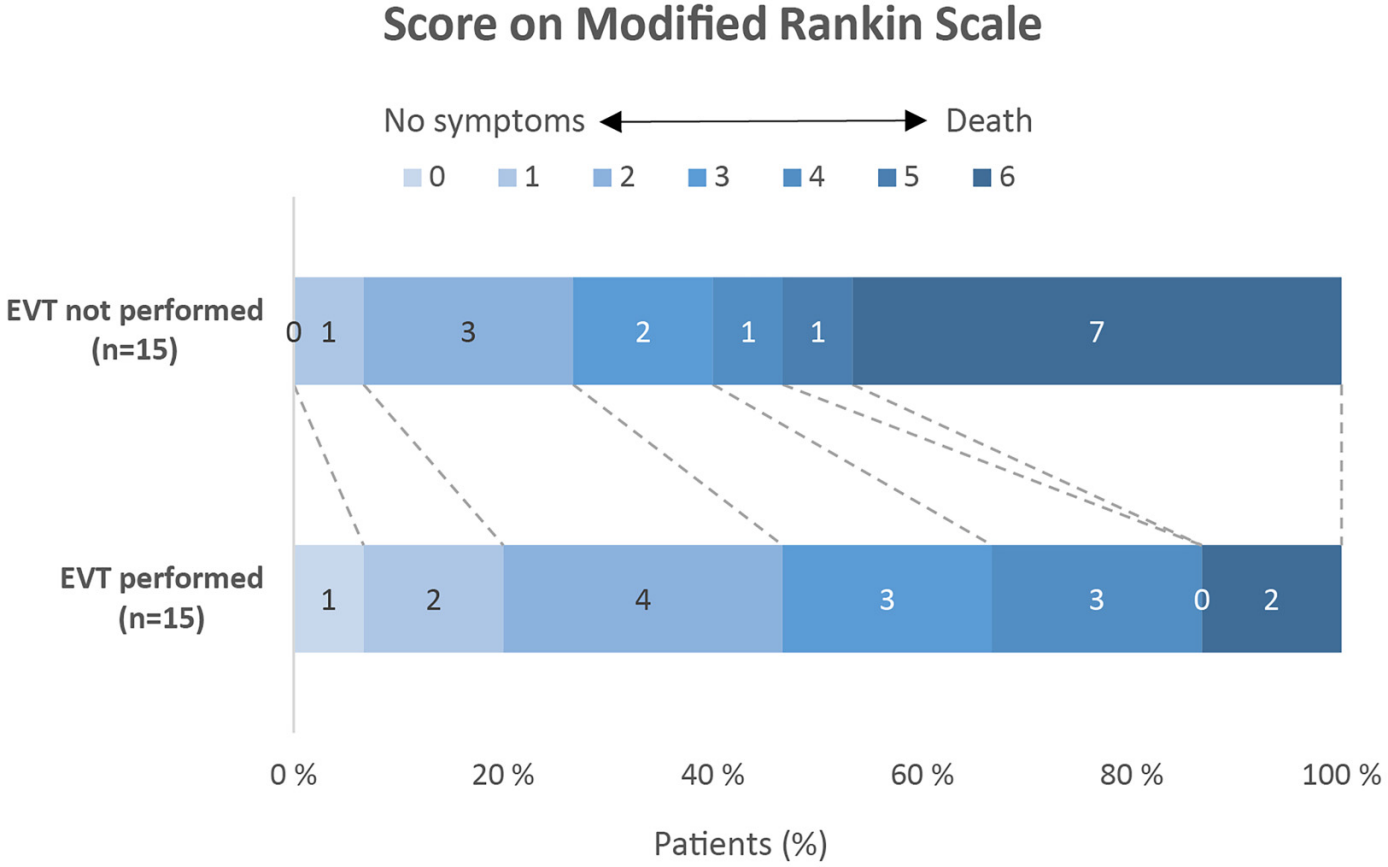
mRS score after 3 months. The mRS scores range from 0 to 6, with 0 indicating no symptoms, 1 no significant disability, 2 slight disability (unable to carry out all pre-stroke activities, but able to look after self without daily help), 3 moderate disability (requiring some help, but able to walk without assistance), 4 moderately severe disability (unable to walk or attend to bodily functions without assistance), 5 severe disability (bedridden, incontinent, requires continuous care), and 6 death. The number of patients with severe disability or death as clinical outcome (defined as mRS 5-6) was significantly higher for the patients with EVT not performed than with EVT performed (*p* = 0.020).

The scores on the mRS after 3 months were registered for all EVT-eligible patients. Among the 15 patients who underwent EVT, seven (47%) were functionally independent (mRS 0-2) after 3 months, while two patients (13%) did not survive. In contrast, of the 15 eligible patients who did not receive EVT, four (27%) were functionally independent after 3 months, one (7%) was severely disabled and seven (47%) did not survive (Figure 2). The incidence of severe disability or death (mRS 5-6) was significantly higher for the patients who did not undergo EVT compared to those who did (Figure 2, *p* = 0.020).

mRS: modified Rankin scale; EVT: endovascular thrombectomy.

### Timelines for transported patients

Twenty-two patients were transported from the primary stroke center to the comprehensive stroke center. The median time interval from stroke symptom onset to arrival at the primary stroke center was 60 min.

EVT was performed for 15 and not performed for seven of these patients after transfer. At arrival, repeated imaging (MRI) was conducted for 12 of the 15 patients who underwent EVT and for six of the seven patients who did not.

The median time intervals were as follows:

- 12 min from arrival to CT imaging at the primary stroke center,
- 3 h and 3 min from CT imaging to arrival at the comprehensive stroke center,
- 19 min from arrival at the comprehensive stroke center to repeated imaging.

**Figure 3.**
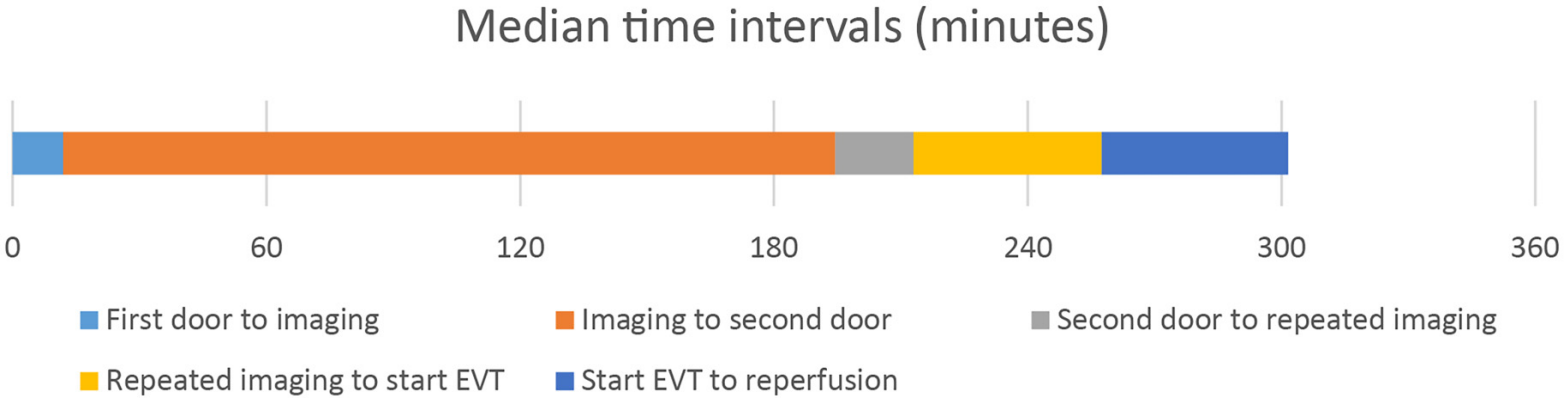
Median time intervals for transported patients. First door: arrival at the primary stroke center; imaging: CT imaging at the primary stroke center; second door: arrival at the comprehensive stroke center; repeated imaging: MRI at the comprehensive stroke center; start EVT: arterial puncture before EVT, reperfusion: brain reperfusion after EVT. The median time intervals were 12 min from first door to imaging, 3 h and 3 min from imaging to second door, 19 min from second door to repeated imaging, 45 min from repeated imaging to start EVT, and 44 min from start EVT to reperfusion. Three patients went directly to EVT with no repeated imaging, see Supplemental Table S2 for details.

CT: computed tomography; MRI: magnetic resonance imaging; EVT: endovascular thrombectomy.

For the patients who underwent EVT, the median time was 45 min from repeated imaging to groin puncture, and 44 min from the start of the EVT procedure to reperfusion (Figure 3).

Three patients went directly to EVT without repeated imaging. For these patients, the median time interval from arrival at the comprehensive stroke center to the start of the EVT procedure was 40 min. Further details are provided in Supplemental Table S2.

## Discussion

In this study, we found that half of the eligible patients did not receive EVT after being transported to a distant comprehensive stroke center, or were not transported at all. The primary reason was that post-transfer MRI scans showed established infarction and too small or no remaining penumbra. This likely resulted from transport times, with a median time exceeding 3 h from CT imaging at the primary stroke center to arrival at the comprehensive stroke center. The delay, which includes decision-making, organizing of air medical services, and patient preparation, was significantly longer than the actual helicopter flight time, which is estimated to be 75 min.

Our study revealed a significant gender disparity in the administration of EVT, with fewer women receiving EVT compared to men (*p* = 0.028). This may indicate that female patients were less likely to undergo EVT compared to their male counterparts. The finding aligns with existing literature that suggests potential gender differences in stroke treatment and outcomes.^22^ Several factors might contribute to this disparity, including potential biases in clinical decision-making, differences in stroke presentation between genders, and other socio-demographic factors. Further research is necessary to understand the underlying causes of this disparity and to ensure equitable access to EVT for all patients, regardless of gender.

The effectiveness of the EVT treatment is highlighted by the results of this study as patients who were not treated with EVT had a significantly higher NIHSS score after 24 h and a significantly higher risk of severe disability or death after 3 months compared to the patients who underwent EVT. Specifically, the 15 patients who did not receive EVT had no initial improvement in NIHSS, and while nearly half of the patients receiving EVT were functionally independent, nearly half of the patients who did not undergo EVT did not survive at the three-month follow-up. Importantly, more than 30% of the transported patients missed the opportunity for EVT due to transport delays, underscoring the critical need for timely intervention.

At initial assessment in the primary stroke centers, 30% of the LVOs were not diagnosed, and half of these patients had a clinical indication for EVT. While CT angiography was performed routinely, CT perfusion was conducted more selectively in 2018. Previous studies have shown that up to 20% of LVOs can be missed using CT angiography alone.^23^ The use of multiphase CT angiography can improve LVO detection rates. In a study by Ospel et al.,^24^ the detection accuracy of medium-vessel occlusions rose from around 60% to nearly 90% with good interrater agreement using multiphase CT angiography. Using CT perfusion also appears to be highly sensitive for identifying LVOs, especially in proximal and medium vessel occlusions.^25^ After the study period, CT perfusion was implemented as part of routine imaging at the primary stroke centers in Agder County. Furthermore, the establishment of local EVT could potentially enhance the detection rate as local radiologists experience the relevance of LVO diagnosis for treatment decisions. Diagnostic accuracy may further improve with advanced imaging techniques and future artificial intelligence diagnostic tools.^26^

International guidelines recommend that at least 90% of patients who meet the institution's selection criteria should be treated with EVT.^27^ However, in our study, only a small proportion of eligible patients actually received EVT, primarily due to transport delays and missed diagnoses. This low treatment rate can lead to poorer outcomes for the overall LVO stroke population, despite excellent results in patients who actually receive EVT.^28^

This study has limitations, including the retrospective reconstruction of EVT eligibility by an expert consensus group, which is not equivalent to decision-making in an acute clinical setting. Furthermore, the study's relatively small sample size and limited time period reduce the statistical power regarding clinical outcomes. However, our study accurately represents a real-world setting by using actual patient data from a geographic area located at a significant distance from the nearest comprehensive stroke center. Additionally, our population-based setting, where there are no other hospitals treating or referring acute stroke patients to the comprehensive stroke center, enhances the generalizability of our findings. Therefore, our setting can serve as a model for hospitals similarly distanced from EVT-capable centers, providing valuable insights into the real-life challenges and potential solutions for improving stroke care in such regions.

Our findings likely reflect challenges shared by other regions and primary stroke centers with long transport distances to comprehensive stroke centers worldwide. The large geographical variation in access to treatment raises questions about the best organization of stroke services. One proposed model is a two-tier system, with top-level comprehensive stroke centers in densely populated areas and EVT-capable stroke centers with more lenient expertise and volume requirements in less densely populated areas.^29^ While these EVT-capable stroke centers may not match the effectiveness of comprehensive stroke centers on an individual level, improved access to rapid treatment could increase EVT eligibility and overall treatment effectiveness on a population level.^30^

Multisociety consensus guidelines recommend that an EVT-capable stroke center should be reachable within 60 min in rural areas.^31^ In line with this, Agder County reorganized stroke services and established a local EVT-capable center in 2019. Since the annual number of EVTs at this center is lower than international recommendations^2,32^ and the interventional radiologists have limited experience, the implementation is supported by a simulation-based quality improvement program with continuous clinical practice monitoring.^33^ The current study will act as a baseline for future research on how the reorganization affects LVO detection rates, timelines, EVT treatment rates, and clinical outcomes.

## Supplemental Material

sj-docx-1-ine-10.1177_15910199241278036 - Supplemental material for Barriers to stroke treatment: The price of long-distance from thrombectomy centers Supplemental material, sj-docx-1-ine-10.1177_15910199241278036 for Barriers to stroke treatment: The price of long-distance from thrombectomy centers by Olav Søvik, Halvor øygarden, Arnstein Tveiten, Martin Wilhelm Kurz, Kathinka Dæhli Kurz, Pål Johan Stokkeland, Hanne Brit Hetland, Hege Langli Ersdal and Per Kristian Hyldmo in Interventional Neuroradiology
