# Supplementary material for: Barriers to stroke treatment: The price of long-distance from thrombectomy centers
Source: Interv Neuroradiol. 2024 Sep 5;32(5):2328–35. doi: 10.1177/15910199241278036 (PMC11571533; doi:10.1177/15910199241278036)
Supplement: Supplementary file 1 [file sj-docx-1-ine-10.1177_15910199241278036.docx]

**Supplemental material:**

**Table S1:** Agder County patients with more than one ischemic stroke admission in 2018

| **Admissions beyond the**  **24-hour time window** | **No LVO diagnosed by expert neuroradiologist** | **No clinical EVT indication** | **Not transferred** | **EVT not performed after transfer** |
| --- | --- | --- | --- | --- |
| 1 | 1 |  |  |  |
|  | 2 |  |  |  |
|  | 1 |  | 1 |  |
|  | 2 |  |  |  |
|  | 2 |  |  |  |
|  | 1 | 1 |  |  |
| 1 |  |  |  | 1 |
|  | 1 |  |  | 1 |
|  | 1 | 1 |  |  |

LVO: Large-vessel occlusion; EVT: Endovascular thrombectomy

Nine of the Agder County patients had two admissions for stroke registered in the Norwegian Stroke Registry in 2018. However, none of these had LVO at more than one admission.

**Table S2:** Number of transported patients with associated time intervals

|  | **Symptom onset to first door** | **First door to imaging** | **Imaging to second door** | **Second door to repeated imaging** | **Repeated imaging to start EVT** | **Start EVT to reperfusion** |
| --- | --- | --- | --- | --- | --- | --- |
| **Minutes**  Median (IQR) | 60 (45-95) | 12 (7-27) | 183 (150-271) | 19 (14-24) | 45 (35-51) | 44 (30-99) |
| **Number of patients** | 22 | 22 | 22 | 18 | 12 | 15 |

n: Number of patients; IQR: Interquartile range; EVT: Endovascular thrombectomy; Symptom onset: Debut of stroke symptoms; First door: Arrival at the primary stroke center; Imaging: CT imaging at the primary stroke center; Second door: Arrival at the comprehensive stroke center; Repeated imaging: MRI at the comprehensive stroke center; Start EVT: Arterial puncture before EVT, Reperfusion: Brain reperfusion after EVT.

If a patient had a stroke during their inpatient stay at the primary stroke center, “symptom onset” was set to the time when the patient was last observed to be well, and “first door” was determined to be the time when the patient’s stroke symptoms were first identified.

Twenty-two patients were transported from the primary stroke center to the comprehensive stroke center. Three of these patients went directly to EVT with no repeated imaging, while one patient were not accepted for EVT without repeated imaging. The remaining 18 patients had repeated imaging on arrival at the comprehensive stroke center. Twelve of these patients underwent EVT after repeated imaging, while six did not. For the three patients who went directly to EVT with no repeated imaging, the median time from second door to start EVT was 40 minutes. A total of 15 transported patients underwent EVT.
